# Using *in situ* management to conserve biodiversity under climate change

**DOI:** 10.1111/1365-2664.12602

**Published:** 2016-01-19

**Authors:** Owen Greenwood, Hannah L. Mossman, Andrew J. Suggitt, Robin J. Curtis, Ilya M. D. Maclean

**Affiliations:** ^1^ Environment and Sustainability Institute University of Exeter Penryn Campus Penryn TR10 9FE UK; ^2^ School of Science and the Environment Manchester Metropolitan University Manchester M1 5GD UK

**Keywords:** adaptive management, biodiversity conservation, climate‐change adaptation, environmental change, global warming, habitat restoration, managed realignment

## Abstract

1. Successful conservation will increasingly depend on our ability to help species cope with climate change. While there has been much attention on accommodating or assisting range shifts, less has been given to the alternative strategy of helping species survive climate change through *in situ* management.
2. Here we provide a synthesis of published evidence examining whether habitat management can be used to offset the adverse impacts on biodiversity of changes in temperature, water availability and sea‐level rise. Our focus is on practical methods whereby the local environmental conditions experienced by organisms can be made more suitable.
3. Many studies suggest that manipulating vegetation structure can alter the temperature and moisture conditions experienced by organisms, and several demonstrate that these altered conditions benefit species as regional climatic conditions become unsuitable. The effects of topography on local climatic conditions are even better understood, but the alteration of topography as a climate adaptation tool is not ingrained in conservation practice. Trials of topographic alteration in the field should therefore be a priority for future research.
4. Coastal systems have the natural capacity to keep pace with climate change, but require sufficient sediment supplies and space for landward migration to do so. There is an extensive literature on managed realignment. While the underlying rationale is simple, successful implementation requires careful consideration of elevation and past land use. Even with careful management, restored habitats may not attain the physical and biological attributes of natural habitats.
5. *Synthesis and applications*. The recent literature provides a compelling case that some of the adverse effects of climate change can be offset by appropriate management. However, much of the evidence for this is indirect and too few studies provide empirical tests of the long‐term effectiveness of these management interventions. It is clear from the existing evidence that some techniques have a higher risk of failure or unexpected outcomes than others and managers will need to make careful choices about which to implement. We have assessed the strength of evidence of these approaches in order to demonstrate to conservation professionals the risks involved.

## Abstract 2

The recent literature provides a compelling case that some of the adverse effects of climate change can be offset by appropriate management. However, much of the evidence for this is indirect and too few studies provide empirical tests of the long‐term effectiveness of these management interventions. It is clear from the existing evidence that some techniques have a higher risk of failure or unexpected outcomes than others and managers will need to make careful choices about which to implement. We have assessed the strength of evidence of these approaches in order to demonstrate to conservation professionals the risks involved.

## Introduction

Over the next 100 years, climate change is likely to become one of the main drivers of biodiversity loss world‐wide (Maclean & Wilson 2011). Conservation policymakers and practitioners thus face the challenge of enhancing the adaptive capacity of biodiversity to climate change (Heller & Zavaleta 2009). However, ecosystems have been modified extensively and it is likely that a substantial proportion of species will be hindered from tracking climate change by their inability to traverse large distances over hostile land cover (Mantyka‐Pringle, Martin & Rhodes 2012). Although numerous species have redistributed towards higher latitudes and elevations (Chen *et al*. 2011), for many the shift has not been fast enough to keep pace with climate change (Menéndez *et al*. 2006). While much discussion of adaptation to climate change has focussed on accommodating or assisting these range shifts, less attention has been given to the alternative strategy of improving species' ability to cope with climate change within their existing range. One means the conservation world has of achieving this is by manipulating habitat conditions to better match species requirements. However, to date, there has been little guidance from scientists about how this can be achieved.

Many species, particularly in Europe and North America, are reliant on habitat manipulation (e.g. Luoto, Pykälä & Kuussaari 2003). It has also been demonstrated that some species can alter their use of habitat in response to variation in climate, for example utilizing cooler habitats more frequently when temperatures are warmer (Suggitt *et al*. 2012). Taken together, these lines of evidence suggest that habitats can be manipulated to buffer species against the adverse effects of climate change. The evidence that such an approach may be effective, while indirect in many cases, is growing. Here we review this evidence. Temperature is not the only component of the climate that is changing, however. Changes in precipitation and, by extension, water availability may have even greater impacts on ecosystems than temperature and indirect impacts such as from sea‐level rise will also be important (IPCC 2013). Our review thus focuses on terrestrial impacts and on three of the major environmental changes associated with climatic change: temperature, water availability and sea‐level rise.

A wide spectrum of site‐based approaches has been proposed to adapt conservation to climate change. However, many are generic, available to conservation managers irrespective of climate change. It is impractical to attempt to cover all techniques for site‐based conservation in a single review, so our review of these wider techniques is limited to a brief synthesis. Our primary focus is on how *in situ* management could be used to manipulate the climatic conditions experienced by organisms. As our aim is to provide guidance for site managers, we also highlight a few instances where localized landscape management, such as catchment hydrology manipulation, could enhance the *in situ* persistence of target species.

## Materials and methods

To identify potential management techniques, we searched Web of Science using terms related to climate change and management (see Appendix S1 in Supporting Information for list), identifying 101 studies as potentially relevant. Each of these was studied and the reference list queried to identify further relevant studies. Any additional studies known to the authors were also included. Our review is based on information in 67 relevant papers identified in this way. Full details of the search methods are provided in Appendix S1.

The strength of evidence for each management technique was assigned a quantitative score using three criteria: (i) the magnitude of the responses reported by each study; (ii) the overall confidence in the documented responses and (iii) the number of studies reporting that management technique. The risk of failure associated with each management technique, both in terms of the risk that the technique is ineffective and in terms of undesirable side effects, was assigned a quantitative score using: (i) the likelihood of an adverse response (assessed using the confidence in reported responses) and (ii) evidence in the wider literature not pertaining to climate change that such management can have undesirable effects. Economic feasibility was not considered. Formal definitions and the methods by which scores were combined are provided in Appendix S1. A full list of potential management techniques is provided in Table 1. Those that have been shown to be consistently ineffective are shown in Table 2.

**Table 1:** Management responses to climate change, with associated effects on the environment and on wildlife. For each response, the strength of supporting evidence and risk of failure is also assessed. Separate assessments for each study are provided in Table S3. Superscript numbers cross‐reference with those in Appendix S2, in which further details are provided

Adverse effect	Management technique	Positive effect on wildlife	Potential adverse effects on wildlife	Strength of evidence	Risk of failure
Warming	Afforestation^1–3^ and abandonment/reduced grazing^4^	Increased/denser vegetation cover reduces maximum temperatures and buffers species against temperature extremes, but may have undesirable effects on non‐target species	Increased resource competition	Moderate/Strong	Medium
	Slope creation/protection^5–7^	Equatorward‐facing slopes accommodate range‐expanding species; poleward‐facing slopes benefit range‐retracting species. Topographic heterogeneity buffers species against adverse effects of climate	Reduced availability of optimal habitat	Strong	Medium
	Woody debris addition^8^	Stabilizes soil temperature and reduces moisture loss benefiting species with high moisture and low temperature requirements	Reduced light availability	Low	Medium
Precipitation change	Altering grazing regimes^9,10^	Livestock exclusion counteracts hydrological effects of increased winter precipitation in California with benefits to plants, amphibians and invertebrates. Increased grazing reduces infiltration and enhancing small‐scale heterogeneity in hydrological conditions, benefiting ephemeral wetland species in the UK. High risk of failure as grazing can have both positive and negative impacts	Reduced grazing may reduce diversity, particularly in areas with productive soils and high rainfall	Moderate	High
	Manipulate water flow with permeable^11^ or impermeable barriers^12^ or drainage control^12,13^	Permeable barriers regulate water flow and create shallow pools. Biological benefits untested. Drain blocking enhances key peatland species. Diverting ditches improves conditions for wet grassland birds	Unknown	Moderate	Low
	Irrigation/spraying^14^	Increases water availability; enhanced amphibian spawning. Expensive	Reduced water availability elsewhere	Strong	Medium
Sea‐level rise	Sea‐defence creation/maintenance^15–19^	Protects coastal habitats from seawater intrusion. Benefits non‐marine species or those with specific salinity/water requirements. Creation of textured surfaces and artificial rock pools create habitat for intertidal organisms. Options for soft‐engineering oyster and mussel beds as offshore barriers. Stabilization/accretion of material on sandy beaches	Altered sediment transport may increase erosion offsite	Strong	Medium
	Stabilization of intertidal and coastal habitat^20,21^	Sediment addition to intertidal habitat increased surface elevation offsetting sea‐level effects with benefits to intertidal communities. Planting/protection of, for example, cordgrass or marram grass stabilizes coastal habitats	Cordgrass is highly invasive, potentially reducing native biodiversity	Strong	Medium
	Defence realignment^22–24^	Intertidal habitat creation. Benefits to waders, saltmarsh plants and benthic invertebrates	Adverse effects unlikely, but benefits depend on shore profile and morphology	Moderate	Medium
	Active management of newly created habitat, including seeding^25^, reprofiling and sediment addition^26^	Ensures newly created intertidal habitat more similar to natural habitat. Increased diversity of benthic invertebrates and saltmarsh plants	Reduces suitability of wader feeding habitat (exposed mud)	Moderate	Low

**Table 2:** Potential management responses to climate change, which have never been shown to work. Superscript numbers cross‐reference with those in Appendix S3, in which further details are provided

Adverse effect	Management technique
Warming	Adding fertilizer to promote vegetation growth^1^
Precipitation change	Keeping rice fields flooded after harvest^2^
	Rewetting soils in old arable fields^3^
Sea‐level rise	Raising areas of substrate for nesting birds^4^

## Management to offset the effects of temperature change

Mean temperatures and the frequency of extreme warm temperature events are both predicted to increase by 2100 (IPCC 2013), with two important implications for wildlife: (i) populations or individuals that fail to track their thermal niche could suffer a reduction in fitness, leaving them more vulnerable to other stressors and (ii) the increasing regularity of extreme events will give populations less time to recover from shocks (Oliver, Brereton & Roy 2013). The principal means of offsetting warming involve manipulation of vegetation and/or topography. Differences in vegetation type and height are well‐established modifiers of the thermal environment. Local temperatures in areas with less vegetation cover are generally cooler during the night and warmer during the day (Suggitt *et al*. 2011) and several studies, particularly on thermophilous insects, demonstrate the importance of these variations in microclimate in determining distribution and abundance (Thomas 1993). For example, for the Glanville fritillary butterfly, the availability of suitable microclimates (as determined by the successional stage of vegetation) is almost twice as strong a predictor of butterfly abundance as regional air temperature (Curtis & Isaac 2015), probably because species can change habitat association in response to ambient temperatures (Suggitt *et al*. 2012). Given that species may shift into relatively cooler habitats in response to warmer temperatures, it would appear axiomatic for land managers to implement management that results in more vegetation cover. However, given that loss of early‐successional habitat has been linked to species declines (e.g. Thomas *et al*. 2004) and that such habitats can be cooler at night, the creation (and maintenance) of thermally diverse habitats remains the current priority in insect conservation (Thomas, Simcox & Hovestadt 2011). Although there is less evidence for taxa other than butterflies, it has been suggested that the thermal properties of microhabitats influence the distribution of a variety of other taxa (e.g. Kearney *et al*. 2007; Barnagaud *et al*. 2013).

In aquatic ecosystems, where fluctuations in temperature are dampened by the higher specific heat capacity of water, a number of studies indicate that the maintenance of riparian shade can reduce temperatures sufficiently to offset the effects of climate change. For example, Broadmeadow *et al*. (2011) demonstrated that even relatively low levels of shade (20–40%) can be effective in keeping summer temperatures below the incipient lethal limit for brown trout *Salmo trutta* L., although *c*. 80% shade would be needed to prevent temperatures exceeding those for optimal growth. While the evidence relates to salmonoid fish in cold‐water streams, there is growing evidence from a broader range of systems (e.g. Mantyka‐Pringle *et al*. 2014; Table 1). Additionally, riparian shading management may also increase bank stability and reduce sediment transport and/or erosion (Pawson *et al*. 2013). This practice is the subject of an increasing number of focussed initiatives world‐wide (Britain, Lenane 2012); California, Stein *et al*. 2013 Other actions to improve water availability in aquatic ecosystems (e.g. artificial wetting; Mitchell 2001) are also likely to reduce the effects of extreme heat.

Topography, particularly the aspect and angle of slopes, controls the amount of radiation received near the Earth's surface and hence exerts strong influences on the temperatures experienced by many organisms, particularly in mid‐latitudes to high latitudes (Table 1). As with vegetation structure, there is much evidence that local variation topography interacts with regional climate to have major influences on species distribution and abundance. For example, many species are restricted to warmer, equatorward slopes at their poleward (cold) range margin (Pigott 1968). Increasing evidence also demonstrates that variations in topographic microclimate can also buffer the effects of climate change (Suggitt *et al*. 2014, 2015; Maclean *et al*. 2015). While the potential to alter topography through management is not well ingrained in conservation practice, there have been notable successes (Table 1). For example, work to restore quarries after mineral extraction (Nature After Minerals 2015), and more specifically the creation of artificial scrapes (e.g. Slater 2014) have been shown to benefit both butterflies and plants. Furthermore, many housing and infrastructure development projects entail the artificial profiling of construction sites, which in some cases has led to successful colonizations of sites previously unimportant for wildlife (e.g. Danahar 2011). Increasingly, developers are required to mitigate or offset the ecological impacts of construction through the creation or restoration of habitats for wildlife (Defra 2013). It is easy to envisage a process whereby topographic variation is deliberately enhanced as part of such activities.

Given the effort and likely expense associated with altering topography or manipulating vegetation, the current advice to land managers remains that the creation of thermally diverse areas can be beneficial in that it can promote population stability, ameliorate the higher and more variable temperatures associated with climate change and is likely to provide habitat for a wider variety of species (Macgregor & van Dijk 2014; Table 1). However, many sites are managed specifically for single species or related species reliant on specific habitat or topographic requirements. In these instances, the creation of more heterogeneous environments would be undesirable if at the expense of reducing the amount of optimal habitat. For example, within the UK, maximizing the availability of warm microclimates could benefit one‐sixth of rarer British butterfly species (Thomas 1993), but this creation of warm microclimates may be detrimental to the remainder. The trade‐off between maintaining species diversity and increasing (general) abundance remains complex and reinforces that research at greater spatial and ecological detail remains a priority to understand the impact of climate change (Kearney & Porter 2009).

## Management to offset the effects of water availability change

Globally, trends in precipitation are not clear‐cut (IPCC 2013) and environmental managers are likely to be faced with the challenge of adapting nature conservation to both wetter and drier conditions, sometimes in the same location at different times of year. Notwithstanding this challenge, there is a substantial precedent in managing landscapes to regulate water supply (Table 1), reduce flood risk (O'Connell *et al*. 2007) and manage water levels to enhance biodiversity (Eglington *et al*. 2010), and thus, there is considerable potential to offset the effects of climatic change on water availability through habitat management.

Broadly, three management approaches have been used to influence water availability (Table 1), although many examples are not specifically associated with adapting nature conservation to climate change. The first entails modifying land use to divert or regulate water supply downstream. In grazing marshes in the East of England for example, artificial shallow drains have been used to divert water to the middle of marshes. This process creates areas of flooding and damp habitat that can potentially provide a mosaic of nesting habitat and profitable feeding areas for breeding waders (Eglington *et al*. 2010). Similarly, Mitchell (2001) manipulated water availability at breeding sites for brown toadlet *Pseudophryne bibronii* Günther in South Australia using portable irrigation sprayers, with improvements in breeding success. The small spatial scale at which most amphibians operate makes them ideally suited to habitat manipulations of this type and there is consequently considerable potential to offset some of the adverse effects of climate change on amphibians through active management (Table 1).

A second approach involves manipulating catchment hydrology to influence water availability upstream. For example, the soil moisture of peatlands in the United Kingdom has been manipulated by blocking ditches. This in turn increases cranefly Tipulidae abundance, particularly in dry years (Table 1). Craneflies are a key herbivore in these habitats and an important prey item for breeding birds, but they are susceptible to drought. The diversion of water (partly to benefit wildlife) can, on occasion, operate on a grand scale. In Florida, for example, there are plans to construct canals and levees to restore the everglades over an area of 47 000 km^2^ (RECOVER 2014).

Lastly, habitat management can be used to manipulate vegetation structure, which in turn influences hydrology by affecting evapotranspiration. For example, Pyke & Marty (2005) showed that cattle grazing offsets the effects of increased winter precipitation on the hydroperiod of ephemeral wetlands by enhancing evapotranspiration, thus improving conditions for endangered invertebrates and amphibians. However, cattle grazing can also have the opposite effect. The depressions created by livestock trampling often accumulate water, and in some instances grazing is used as a means of ensuring conditions remain suitably wet (Maclean *et al*. 2012; Scott *et al*. 2012). Thus, the effects of grazing on hydrological conditions are not necessarily predicable and site‐level knowledge or experimentation may be essential for successful conservation outcomes.

This latter finding serves to illustrate one of the challenges faced by managers: namely what to do when. Arguably the most important consideration will be what changes are expected. Where reductions in water availability are forecasted, creating wetter conditions is likely to be beneficial and vice versa. Where greater variability is predicted, the creation of a stable water supply is likely to be desirable. A means of achieving greater stability is through the creation of permeable timber barriers, artificial diversion ponds and careful positioning of woody debris in streams, all techniques which have been used to attenuate run‐off during periods of high rainfall (Table 1). Where there is uncertainty surrounding the availability of water, techniques that enhance heterogeneity in water availability are likely to be the most effective as they will increase the likelihood that suitable conditions for target species exist. Management techniques for achieving this include the creation of shallow scrapes and pools using heavy construction plant machinery (Natural England 2010) or encouraging low‐density livestock grazing and trampling in marshes, fens and wet meadows (Tesauro & Ehrenfeld 2007). Bunding ditches (or diverting them to increase drainage in areas where susceptible to undesirable flooding) should also be considered as interventions in wet grassland, peatland and mire systems (Hopkins *et al*. 2007). It should be noted, however, that grazing can also have adverse effects in some ecosystems, particularly drier systems, or fail to have desired benefits (Lunt, Jansen & Binns 2012) and increasing heterogeneity may reduce the availability of optimal habitat. Any changes in grazing regimes or other management techniques implemented to increase heterogeneity should thus proceed with caution.

## Management to offset the effects of sea‐level rise

Global sea levels rose by approximately 0·19 m between 1901 and 2010 (Hay *et al*. 2015) with predicted rises of 0·25–1 m over the 21st century (IPCC 2013). Rising sea levels affect the extent and quality of coastal habitats through erosion and changes in niche availability and increase the vulnerability of inland habitats to seawater flooding. There are particular problems where coastal development and construction of hard defences prevent landward migration of habitats, resulting in them being squeezed between a fixed landward boundary and rising sea levels (Morris *et al*. 2004). While this review deals with *in situ* management in response to these threats, it is worth emphasizing that such management should sit alongside landscape approaches, because even modest coastal development can alter natural coastal dynamics over hundreds of kilometres (Hapke, Kratzmann & Himmelstoss 2013).

Appropriate *in situ* management to offset the effects of sea‐level rise depends mainly on the habitat type in question. Freshwater and brackish habitats, such as saline lagoons, require protection from tidal inundation because species are vulnerable to increases in salinity, which can lead to shifts in community composition (Tate & Battaglia 2013). Where landward retreat of these habitats is not possible due to adjacent land use, protection from saline flooding by the maintenance of hard or natural defences (e.g. sand or shingle barriers) is likely to be most effective. For example, sea walls at RSPB Titchwell, Norfolk, UK, were replaced or strengthened to protect important freshwater habitats, as part of a package of measures aimed at adapting the reserve to rising sea levels (RSPB 2013). Given the conservation value of these specialist communities (Beer & Joyce 2012) and their vulnerability to sea‐level rise (Spencer & Brooks 2012), investment to maintain defences may be justified.

Rocky intertidal habitats are among the most vulnerable to rises in sea level because many are backed by steep inclines (such as hard cliffs) and are thus unable to retreat landward (Jackson & McIlvenny 2011). Two forms of management are likely to be particularly effective. First, the creation of hard and rock‐armoured defences, such as breakwaters, gabions and offshore barriers, can be used to absorb wave energy and reduce local erosion (French 2001) and are colonized by intertidal organisms. However, intertidal communities on existing hard defences are less diverse than those on natural rocky shores because the defences lack environmental heterogeneity, tending to be smooth and steeply grading (Table 1). Creation of microhabitat features (e.g. shaded vertical surfaces and water‐retaining features that mimic rock pools) increases the diversity of algal and macrobenthos communities and increases the potential for artificial barriers to compensate for loss of existing rocky intertidal habitat (Table 1). An alternative approach is to promote ecologically engineered offshore barriers, such as those created by reef‐building oysters and mussels (Borsje *et al*. 2011). These can attenuate wave energy and stabilize intertidal flats behind them, although their effectiveness may be limited in high‐energy environments (Table 1). Oyster reefs have declined by 85% over the past 100 years (Beck *et al*. 2011), and the creation of ecologically engineered reefs has the dual benefit of increasing habitat extent and providing a self‐sustaining barrier that can keep pace with sea‐level rise (Rodriguez *et al*. 2014). The decision as to which type of barrier to create depends on whether the goal is to create a specialist ecological community (ecologically engineered reef), or provide suitable habitat for a wider algal and macrobenthos community (artificial barriers).

Soft‐sediment intertidal habitats are able to accrete vertically and maintain their elevation with respect to rising sea levels if there is a sufficient supply of sediment and conditions are suitable for settlement (Krauss *et al*. 2014). Structures such as groynes and brushwood fences have been used to interrupt the movement of sediment and encourage local deposition, therefore increasing habitat extent by widening beaches (Table 1). However, if insufficient sediment is available to maintain habitat extent, additional material can be added to the system. For example, material from dredged sites can be added to beaches or eroding saltmarshes to increase the width and/or surface elevation, which may have the added benefit of increasing plant above‐ground biomass, which in turn can stabilize the saltmarsh surface (Table 1). The source of the sediment for such nourishment schemes is an important factor. Fine‐grained material is more likely to be resuspended and washed away and the form of benthic invertebrate communities is highly dependent on the grain size of the added material (Bolam & Whomersley 2005; Table 1).

Creation of new coastal habitats adjacent to existing ones is likely to be the most effective long‐term option. Managed realignment, where sea defences are relocated landward and the old, seaward defences are breached to allow tidal inundation (French 2006), is the most commonly used method to create intertidal flats and saltmarshes. While not strictly *in situ* management, it often within the remit of a site manager to consider such an option and we therefore provide a brief overview of its efficacy. The most important factor in the success of these schemes is the surface elevation of the site, since this determines the colonization and subsequent composition of communities. Most sites selected for managed realignment are low‐lying with respect to sea level (Crooks *et al*. 2002). This maximizes the length of time the habitat remains unvegetated and thus suitable feeding habitat for wading birds (Table 1), but is not desirable if the aim is to quickly establish vegetated saltmarsh (Garbutt *et al*. 2006). While benthic infaunal and saltmarsh plant species can often colonize quickly (Mossman *et al*. 2012), natural communities can be more difficult to recreate (Mossman, Davy & Grant 2012). Artificial planting of rare species accelerates vegetation development, and may be particularly beneficial if the plant species host rare invertebrates (Woodell & Dale 1993). Plant colonization may be constrained by poorly drained and oxygenated sediments (Mossman, Davy & Grant 2012), which may be improved by the establishment of effective creek networks (Crooks *et al*. 2002) or the creation of more varied topography through constructing raised and lowered areas (Table 1). The grazing of saltmarshes can also generate habitat heterogeneity and may be particularly desirable when vegetation is dominated by invasive high‐marsh grasses (Bos *et al*. 2002). In these situations, extensive grazing can increase plant diversity and create habitat more suitable for waterfowl, potentially mitigating for some sea‐level‐induced impacts (Clausen, Stjernholm & Clausen 2013).

## General *in situ* management techniques

In addition to manipulating environmental conditions, there are several more general methods that have been used to enhance the capacity for biodiversity to cope with climate change (see, e.g., Macgregor & van Dijk 2014). At the most generic level this may simply involve reducing other threats. The general contention is that, by reducing or preventing other threats to biota, target wildlife is better able to cope with climate change. Although it can be assumed that ameliorating the risk from these other threats will benefit a species' climate response, direct evidence of this occurring in practice has been more forthcoming for some threats than others. Interactions with pest species have been particularly well documented and there is a substantial amount of evidence that exposure to pest species makes affected species more vulnerable to drought‐induced water stress (Breshears *et al*. 2005), while also impeding the recovery of forests from extreme storm events (Pawson *et al*. 2013). The compounding effects of species invasions and climate change are also well documented, but most of the evidence for the utility of this approach is mixed and context‐dependent, primarily because the evidence for competition‐related declines is similarly conflicting. In the UK for example, ‘non‐native’ plants have limited negative impact on native diversity (Thomas & Palmer 2015), but in the Alps, high‐altitude plants are being out‐competed by low‐altitude plants (Gottfried *et al*. 2012), and here the lack of an alternative habitat (upslope) strengthens the case for interventions to defend what climatically suitable habitat remains. The realities of conservation funding mean that attention in this area is focussed on those species with the highest economic impact, and thus, evidence we have for the efficacy of invasion control is similarly biased. However, there are cases where the increased prevalence of ‘non‐native’ species interacts with climatic conditions to compound the adverse effects. For example, vigorous, competitive invaders such as *Rhododendron ponticum* are likely to reduce understorey microclimatic heterogeneity and floating Cyanobacteria can lead to the loss of cold‐water refugia as a result of hypolimnetic anoxia (Havens 2008).

The maintenance of genetic, species or functional diversity within ecosystems (see Folke *et al*. 2004 for a detailed review), has also been advocated, primarily for the purpose of bet‐hedging: more diverse systems are better positioned to withstand climate change. For example, the effects of extreme drought on plant communities are patchy, affecting some species more than others (Buckland *et al*. 1997). Consequently, maintaining the diversity of these plant communities ‘bet hedges’ that those species that are more tolerant or resistant to drought will be conserved (Di'az & Cabido 2001). The same principle has also been proposed at the genetic level, where populations with more genetic diversity are often found to be more resistant or resilient to extreme climatic events (Jump & Peñuelas 2005). While the general applicability of the ‘maintaining diversity’ approach is at least partially supported by evidence that management to improve diversity in one particular taxon or group often benefits diversity in other groups (Maskell *et al*. 2013), the underlying rationale is at best equivocal. One of the key reasons why increased diversity has been suggested to increase resilience is based on the concept of functional redundancy: more diverse ecosystems are assumed to be better able to maintain function even when some species are lost. Nonetheless, even in diverse systems, the loss of a single species can lead to major changes in ecosystem function (Di'az & Cabido 2001). The opposing side of this argument is that protecting the natural function of ecosystems, species and communities will enhance their capacity to cope with climate change. These processes can be biological (e.g. pollination, dispersal, succession of vegetation) or physical (e.g. erosion and deposition, river migration). A good example of the benefits of maintaining natural processes is the managed realignment of coasts already discussed. However, the approach has been applied more widely and often has multiple benefits. The retention of deadwood and/or debris in forests, for example, both improves the diversity of saproxylic invertebrates (Pawson *et al*. 2013) and offers greater diversity of microhabitats for other potential occupants (Hobson & Mickleburgh 2008). This serves to illustrate a more general point: some (e.g. Bellard *et al*. 2012) have argued that our current knowledge of the impacts of climate change is highly disparate and uncertain. In such situations, ‘no regrets’ techniques are likely to be the most sensible to adopt.

## Conclusions

The threats of climate change to biodiversity are driving changes in recommended conservation practice. However, the majority of recommendations thus far focus on the broader landscape level, for example by enhancing connectivity or increasing the number or size of reserves (Heller & Zavaleta 2009). Surprisingly, despite the extent to which current conservation management practice alters local environmental conditions, the use of management as a tool for manipulating these conditions has rarely been recommended as a means of helping species cope with climate change, except as a means of countering sea‐level rise.

While empirical evidence for the effects on biota of these management actions is in its early stages, it is clear from the evidence already available that, in some circumstances, there is a compelling case for management. Nonetheless, some techniques have a higher risk of failure or unexpected outcomes than others. We have assessed the strength of evidence of a selection of the approaches (Table 1) in order to provide an indicative idea to conservation professionals of the likely effectiveness of a given approach. We also assess the risk of failure, as some techniques may have undesirable effects and provide a list of those techniques that are unlikely to work (Table 2). There will also be inherent trade‐offs: for example, prioritizing heterogeneity will come at the expense of some ‘optimal’ habitat for species. Allowing taller vegetation to establish will reduce light availability and provide a higher degree of competition (WallisDeVries & Van Swaay 2006). The degree to which one strategy or another is preferable will also depend on the time period over which it is enacted, with techniques to protect existing biota at a site more attractive in the short term, but accommodation or even encouragement of change likely to be required in the long term (Rannow *et al*. 2014).

Overall, however, replicated and monitored local manipulations of habitat that ascertain the efficacy of management actions are rather scarce. Perhaps one of the reasons why such case studies are lacking is the weak implementation of adaptive management (e.g. Mitchell *et al*. 2007). In a technical sense, this entails manipulating a system in order to improve understanding and hence manage it more effectively. It is intended to be a structured, iterative process that leads to robust decisions in the face of uncertainty. In reality, however, it is often taken to mean that managers retain flexibility and respond as situations develop (e.g. Mitchell *et al*. 2007) and some argue that the phrase ‘adaptive management’ is usually used to disguise weak conservation practices (e.g. Sutherland 2006). Furthermore, even if applied correctly, the approach relies on there being measurable ecological responses to management that can be distinguished from other factors (Oliver & Morecroft 2014). Consequently, irrespective of whether management is being carried out adaptively or proactively, there is much need for well‐documented examples of habitat manipulations carried out in ways that permit their effectiveness to be established. It is thus important to document failure as well as success. It is likely that future efforts to safeguard biodiversity against the effects of climate change will require a rich variety of approaches. It is our belief that the deliberate manipulation of environmental conditions through habitat management should be considered as part of the suite of options available and the effectiveness of such actions adequately tested and documented.

## Supporting information


**Table S1.** Scheme for scoring the strength of evidence from individual studies.

**Table S2.** Scheme for scoring the strength of evidence from multiple studies.

**Table S3.** Assessment of strength of evidence and risk of failure associated with each study.

**Appendix S1.** Details of systematic literature review.

**Appendix S2.** Additional information and references associated with Table 1.

**Appendix S3.** Additional information and references associated with Table 2.
