# Supplementary material for: Using in situ management to conserve biodiversity under climate change
Source: J Appl Ecol. 2016 Jan 19;53(3):885–94. doi: 10.1111/1365-2664.12602 (PMC4991270; doi:10.1111/1365-2664.12602)
Supplement: Supplementary file 1 — Table S1. Scheme for scoring the strength of evidence from individual studies. [file JPE-53-885-s001.docx]

Table S1. Scheme for cross-tabulating scores associated with the magnitude of a response and the confidence in the response, to derive overall scores of the strength of evidence associated with each study.

|  | | Confidence in response | | |
| --- | --- | --- | --- | --- |
|  |  | High | Medium | Low |
| Magnitude of response | High | Strong | Moderate | Weak |
|  | Medium | Strong | Moderate | Weak |
|  | Low | Moderate | Weak | Weak |
