# Supplementary material for: Using in situ management to conserve biodiversity under climate change
Source: J Appl Ecol. 2016 Jan 19;53(3):885–94. doi: 10.1111/1365-2664.12602 (PMC4991270; doi:10.1111/1365-2664.12602)
Supplement: Supplementary file 2 — Table S2. Scheme for scoring the strength of evidence from multiple studies. [file JPE-53-885-s002.docx]

Table S2. Scheme for scoring the strength of evidence for management techniques tested in multiple studies.

|  | | Number of studies | | |
| --- | --- | --- | --- | --- |
|  |  | 1 | 2-3 | 3+ |
| Median strength of evidence for each study | Strong | Strong | Strong | Strong |
|  | Moderate/Strong * | - | Strong | Strong |
|  | Moderate | Moderate | Moderate | Strong |
|  | Weak/Moderate * | - | Moderate | Moderate |
|  | Weak | Weak | Weak | Moderate |

*In instances where scores are derived from more than one study, the median may fall between categories, for example when one study has a score of high, and another of medium.
