# Supplementary material for: Using in situ management to conserve biodiversity under climate change
Source: J Appl Ecol. 2016 Jan 19;53(3):885–94. doi: 10.1111/1365-2664.12602 (PMC4991270; doi:10.1111/1365-2664.12602)
Supplement: Supplementary file 3 — Table S3. Assessment of strength of evidence and risk of failure associated with each study. [file JPE-53-885-s003.docx]

**Table S3**. Management responses to climate change, with associated effects on the environment and on wildlife. For each individual study, the strength of supporting evidence and risk of failure associated with each study and based on evidence in the wider literature is also assessed. For risk of failure, the criteria met are indicated using the numbering system shown in Appendix S1. Further details and a full list of references are provided in Appendix S2.

| **Adverse effect** |  | **Study** | **Strength of evidence from study** | **Risk of failure (criteria met)** | **Risk of failure derived from wider literature** |
| --- | --- | --- | --- | --- | --- |
| Warming | Afforestation and abandonment / reduced grazing | Ellis *et al*. (2010) | Moderate | Low | Medium (iii,iv) |
|  |  | Scheffers *et al*. (2014a) | Strong | Medium (ii) |  |
|  |  | Scheffers *et al*. (2014b) | Strong | Low |  |
|  |  | Hardwick *et al*. (2015) | Moderate | Low |  |
|  |  | Suggitt *et al*. (2012) | Moderate | Medium (i,ii) |  |
|  |  | Broadmeadow & Nisbet (2004) | Strong | Low |  |
|  |  | Moore, Spittlehouse & Story (2005) | Moderate | Medium (ii) |  |
|  |  | Whitledge *et al*. (2006) | Low | Low |  |
|  |  | Malcolm *et al.* (2008) | Moderate | Medium (ii) |  |
|  |  | Broadmeadow *et al.* (2011) | Strong | Low |  |
|  |  | Parkyn *et al*. (2003) | Low | Medium (ii) |  |
|  |  | McCormick & Harrison (2011) | Strong | Medium (ii) |  |
|  |  | Thomas *et al*. (1998) | Strong | Medium (ii) |  |
|  |  | Davies *et al*. (2006) | Moderate | Medium (ii) |  |
|  |  | Suggitt *et al*. (2012) | Moderate | Medium (ii) |  |
|  | Slope creation / protection | Lawson *et al*. (2012) | Strong | Medium (ii) | Medium (iii,iv) |
|  |  | Bennie *et al*. (2013) | Strong | Medium (ii) |  |
|  |  | Ashcroft, Chisholm & French (2009) | Strong | Medium (ii) |  |
|  |  | Bennie *et al.* (2008) | Strong | Medium (ii) |  |
|  |  | Suggitt *et al*. (2014) | Strong | Medium (ii) |  |
|  |  | Suggitt *et al.* (2015) | Strong | Medium (ii) |  |
|  |  | Bennie *et al.* (2006) | Moderate | Medium (ii) |  |
|  | Debris addition | Haskell *et al.* (2012) | Moderate | Medium (ii) | Medium (iii,iv) |
|  |  | Owens *et al.* (2008) | Low | Low |  |
|  |  | Wanger *et al.* 2009 | Low | Low |  |
|  |  | Patrick *et al.* 2006 | Low | Low |  |
|  |  | Semlitsch *et al.* 2009 | Low | Low |  |
| Precipitation change | Altered grazing regimes | Pyke & Marty (2005) | Strong | Low | High (iii,iv,v,vi) |
|  |  | Moreira & Russo (2007) | Moderate | Low |  |
|  |  | Scott *et al.* (2012) | Strong | High(i,ii) |  |
|  |  | Maclean *et al.* (2012) | Strong | High (ii) |  |
|  | Manipulate water flow with permeable or impermeable barriers or drainage control | Richter *et al.* (2003) | Moderate | Low | Low |
|  |  | Wilkinson, Quinn & Welton (2010) | Strong | Low |  |
|  |  | Nicholson *et al.* (2012) | Strong | Low |  |
|  |  | Caroll *et al.* (2011) | Strong | Low |  |
|  |  | Eglington *et al.*( 2008) | Moderate | Low |  |
|  |  | Eglington *et al.* (2010) | Moderate | Low |  |
|  |  | Eglington *et al*. (2009) | Moderate | Low |  |
|  | Irrigation/Spraying | Mitchell (2001) | Moderate | Medium (ii) | Medium (iii) |
|  |  | Shoo (2011) | Moderate | Medium (ii) |  |
|  |  | Krajick (2006) | Low | Low |  |
| Sea- level rise | Sea-defence creation / maintenance | Airoldi *et al.* (2005) | Moderate | Medium (ii) | Medium (iii,iv) |
|  |  | Richards *et al.* (2008) | Moderate | Medium (ii) |  |
|  |  | Firth *et al.* (2013) | Strong | Medium (ii) |  |
|  |  | Chapman & Blockley (2009) | Strong | Medium (ii) |  |
|  |  | Firth *et al.* (2014) | Strong | Medium (ii) |  |
|  |  | Green, Chapman & Blockley (2012) | Strong | Medium (ii) |  |
|  |  | Piazza, Banks & La Peyre (2005) | Moderate | Medium (ii) |  |
|  |  | Borsje *et al*. (2011) | Strong | Medium (ii) |  |
|  |  | Meyer, Townsend & Thayer (1997) | Strong | Medium (ii) |  |
|  | Stabilisation of intertidal and coastal habitat | Hanley *et al.* (2014) | Strong | Medium (ii) | Medium (iii), (iv) |
|  |  | Webb, Oliver & Pik (2000) | Strong | Medium (ii) |  |
|  |  | Nehring & Hesse (2008) | Strong | Medium (ii) |  |
|  |  | Mendelssohn & Kuhn (2003) | Strong | Low |  |
|  |  | Lowe *et al.* (2000) | Moderate | Medium(ii) |  |
|  | Defence realignment | Atkinson *et al.* (2004) | Strong | Low | Medium (iv) |
|  |  | Badley & Allcorn (2006) | Moderate | Low |  |
|  |  | Mazik *et al.* (2010) | Moderate | Low |  |
|  |  | Morris (2013) | Moderate | Medium (ii) |  |
|  |  | Craft *et al.* (1999) | Moderate | Medium (ii) |  |
|  |  | Mossman *et al.* (2012) | Strong | Medium (ii) |  |
|  |  | Wolters *et al.* (2008) | Low | Low |  |
|  |  | Mossman, Davy & Grant (2012) | Low | Medium (ii) |  |
|  |  | Verbeek & Storm (2001) | Moderate | Medium (ii) |  |
|  |  | Doherty & Zedler (2015) | Strong | Low |  |
|  | Active management of newly created habitat, including seeding, re-profiling and sediment addition | Zedler, Callaway & Sullivan (2001) | Moderate | Low | Low |
|  |  | Garbutt *et al.* (2006) | Moderate | Low |  |
