# Supplementary material for: Using in situ management to conserve biodiversity under climate change
Source: J Appl Ecol. 2016 Jan 19;53(3):885–94. doi: 10.1111/1365-2664.12602 (PMC4991270; doi:10.1111/1365-2664.12602)
Supplement: Supplementary file 4 — Appendix S1. Details of systematic literature review. [file JPE-53-885-s004.docx]

**Appendix S1; Details of systematic literature review**

To identify potential management techniques, we searched Web of Science for studies associated with climate change and management, covering the entire period 1900-2014. The following broad search terms associated with climate change and management types were used: (i) *climat* chang** and *manag**, (ii) *habitat stabil** and *climat* chang**, (iii) *graz** and *climat* chang*,* (iv) *invasive removal* and *climat* chang** and (iv) *manag* realignment.* Querying the topic of each paper using these search terms returned a total of 50,875 paper titles, some of which were duplicates. Screening of article titles reduced this number to 721, and subsequent screening of article abstracts left a total of 101 papers that were potentially relevant. Each of these 101 papers was reviewed in detail and the reference list queried to identify further potentially relevant papers and any additional studies known to the authors were also included. A total of 62 relevant papers, upon which our review is based, was identified in this way.

**Assessing the strength of evidence**

The strength of evidence for each management technique was assigned a quantitative score using three criteria: (1) the magnitude of the responses reported by each study, (2) the overall confidence in the reported responses, and (3) the number of studies reporting that management technique. For each study, we first assigned scores for magnitude of responses as follows: a score of ‘high’ was assigned where the mean effect of management across studied organisms or locations resulted in a measured improvement of greater than 25% in the desired biological outcome (e.g. species richness or abundance); a score of ‘medium’ was assigned when the mean improvement was 10-25% and a score of ‘low’ was assigned when the mean improvement was >0-10%. Management techniques in which adverse responses were generally reported (i.e. the overall score was negative) are not included in Table 1.

Our assignment of scores for confidence in results are based on the authors of that study’s own judgement, supplemented by analyses of confidence intervals in instances where these were reported or could be derived from e.g. measurements of standard error. ‘High’ confidence was assigned when 95% confidence intervals did not overlap with zero and/or when the authors’ ascribed high confidence when discussing their results. ‘Medium’ confidence was assigned when 80% confidence intervals did not overlap with zero and/or when the authors’ highlighted a few caveats when discussing their results. ‘Low’ confidence was assigned when 50% confidence intervals overlapped with zero or where the authors’ present plausible alternative explanations for their results. In instances where analyses of confidence intervals and the authors’ discussion yielded conflicting scores, the lower score was used. For each study, the scores for the magnitude and confidence were cross-tabulated (see Table S1) to derive an overall score for each study.

We do not attempt to formally quantify the confidence associated with data from multiple studies using meta-regression techniques, as the effect being measured in each study often differs and it is thus erroneous to assign higher weighting to studies with larger sample sizes. However, based on the simple premise that multiple lines of evidence for a positive effect of a management technique, provides stronger evidence that a particular management technique will be effective when applied to un-studied organisms, where more than one study investigates the effects of a management technique, we calculated a median score across studies and cross-tabulated this against the overall number of studies to derive an overall score of the strength of evidence associated with each management technique (Table S2)

**Assessing the risk of failure**

The risk of failure associated with management technique was assigned a quantitative score using: (1) the likelihood of an adverse response or the intervention being ineffective (assessed using the confidence intervals surrounding each study’s assessment of the impact of a management activity on flora or fauna); and (2) evidence in the wider literature not pertaining to climate change, that such management can have adverse effects. As the financial risks of an action is influenced strongly factors such as location, available budgets and the particular features of landscape or site, and site managers will be in the best position to asses this on a case-by-case basis, economic feasibility was not considered.

Our assignment of scores for risk of failure associated with each study was again based on the study author’s/authors’ own judgement, supplemented by analyses of confidence intervals in instances where these were reported or could be derived from e.g. measurements of standard error. High risk of failure was assigned when 50% confidence intervals overlapped with zero and/or when the authors’ indicate a high likelihood of failure when discussing their results. Medium risk of failure was assigned when measured 80% confidence intervals overlapped with zero and/or when the authors’ highlighted some risk of failure when discussing their results. Low risk of failure was assigned when 80% confidence intervals did not overlap with zero or when the authors do not indicate any reason for failure.

However, in some instances, there was deemed to be a high risk of failure, even when individual studies associated with climate change and management do not allude to this, because the wider literature not pertaining to climate change either indicates directly that: a) such management can have adverse effects, or b) that the converse of a particular technique can have a beneficial effect. For example, reduced grazing generally has a high risk of failure because increased grazing has been shown to have benefits to biodiversity in some ecosystems. Our assignment of scores for a general risk of failure were derived as follows: a ‘high’ risk of failure was assigned when either there was (i) evidence from a wide range of study systems, or from the specific study system concerned, that the management technique in question has undesirable effects; or (ii) evidence from a wide range ( > 5) of study systems, or from the specific study system concerned, that the converse or cessation of the management technique in question has beneficial effects. A ‘medium’ risk of failure was assigned when either there was: (i) evidence from 2 to 5 five study systems, but not the study system concerned, that the management technique in question has undesirable effects; or (ii) evidence from 2 to 5 study systems, but not the specific study system concerned, that the converse or cessation of the management technique in question has beneficial effects. In all other instances a score of ‘low’ was assigned. The median score derived from individual studies, and the general score derived from wider literature, were both calculated for each management technique and that which was higher used as our final score. A summary of the criteria used to assess the risk of failure is provided below:

**High:**

1. 50% Confidence intervals overlap with zero; or
2. Authors indicate high risk of failure when discussing their results; or
3. Evidence from study system in wider literature that management technique has undesirable effect; or
4. Evidence from >5 other study systems that management technique has undesirable effect; or
5. Evidence from study system in wider literature that converse of management technique has desirable effect; or
6. Evidence from >5 other study systems that converse of management technique has desirable effect.

**Medium:**

If criteria for high are not met; and

1. 80% Confidence intervals overlap with zero; or
2. Authors indicate some risk of failure when discussing their results; or
3. Evidence from 2-5 other study systems that management technique has undesirable effect; or
4. Evidence from 2-5 other study systems that converse of management technique has beneficial effect

If criteria for neither High nor Medium are met, then the risk is low
