# Supplementary material for: Using in situ management to conserve biodiversity under climate change
Source: J Appl Ecol. 2016 Jan 19;53(3):885–94. doi: 10.1111/1365-2664.12602 (PMC4991270; doi:10.1111/1365-2664.12602)
Supplement: Supplementary file 5 — Appendix S2. Additional information and references associated with Table 1. [file JPE-53-885-s005.doc]

**Appendix S2: Additional information and references associated with Table 1**

**Temperature Increase**

*Afforestation*

1. Several woodland butterfly species shift to cooler, closed habitats in hot years (Suggit*t et a*l. 2012) similar to those achieved by afforestation. However closed canopies may alter light availability leading to change in floral composition.
2. Tropical microhabitats provided by afforestation buffer mammals, frogs and lizards against temperature extremes (Elli*s et al*. 2010; Scheffer*s et a*l. 2014a; b; Hardwic*k et a*l. 2015). However, difficult to recreate suitable microhabitat through active management.
3. There is evidence from a range of studies that riverine shading reduces water temperatures and the frequency with which thermal thresholds for fish are exceeded (Broadmeadow & Nisbet 2004; Moore, Spittlehouse & Story 2005; Whitledg*e et a*l. 2006; Malcol*m et a*l. 2008; Broadmeado*w et a*l. 2011). Parkyn *et al.* (2003) review the effectiveness of nine schemes in New Zealand and show canopy closure is required before beneficial effects are manifested, hence time-scales required can be long. McCormick & Harrison (2011) found that dense riparian tree canopy can have a negative effect on salmonid population via trophic interactions, making this a moderately risky technique.

*Abandonment*

1. Evidence from several studies on butterflies show they demonstrate preference for longer sward and/or reduced bare ground cover in warmer years or regions (Thoma*s et a*l. 1998; Davie*s et a*l. 2006; Suggit*t et a*l. 2012) resulting from abandonment. There is a risk that longer sward may reduce light availability and increase nutrient competition for host plants. Late-successional habitats unlikely to be in short supply.

*Slope creation/protection*

1. Provision of equatorward facing slopes facilitates range-expanding Silver-spotted skipper butterfly through increased availability of warmer microclimates (Lawso*n et a*l. 2012; Benni*e et a*l. 2013). The drawback is the potential reduction in availability of cool microclimate for species already present.
2. Poleward facing slopes may benefit range-retracting rainforest species and Alpine grasses as there is less long-term warming on slopes exposed to cool southerly winds in Australia (Ashcroft, Chisholm & French 2009). Effects on microclimate of slope aspect well understood (e.g. Benni*e et a*l. 2008). There is a small risk of limiting availability of warm microclimates for colonising species.
3. Persistence of butterfly and high plant species threatened by climate change is generally higher in areas with high topographic heterogeneity (Suggit*t et a*l. 2014, 2015). However, applied blindly this management may reduce the area of optimal microclimate. Chalk grassland swards on steeply facing sloping are more resistant to invasion by competitive grass species than those on flatter sites due to phosphorus limitation (Benni*e et a*l. 2006)

*Debris addition*

1. Woody debris addition stabilises soil temperature and reduces moisture loss during lakeshore restoration projects, increasing overall survival of plants (Haskel*l et a*l. 2012). May limit light availability to smaller plants if over-applied. Two studies, from the USA (Owens et al. 2008) and Indonesia (Wanger et al. 2009) found that adding coarse woody debris to forest floors had no effect amphibian species richness or overall abundance. Two studies (Patrick *et al.* 2006; Semlitsch *et al.* 2009) show that most amphibian species exhibit no significant difference in abundance in clear-cuts with woody debris removed or retained.

**Rainfall change**

*Altering grazing regimes. Livestock exclusion*

1. Cattle exclusion counteracts hydrological effects of increased winter precipitation (Pyke & Marty 2005). This limits the inundation period increase resulting from precipitation increase in Californian temporary pools. Exclusion benefits invertebrates and amphibians in these pools, as they may fail to develop to maturity with extended inundation periods. Reduced grazing may result in vegetation succession and increased chance of negative effects on species from wild fires (Moreira & Russo 2007). However, high risk of failure as enhanced grazing is beneficial to ephemeral wetland species in other locations (Scot*t et a*l. 2012).
2. Models suggest increased grazing could counteract effects of decreased temporary pool habitat water availability in Cornwall through summer (Maclea*n et a*l. 2012; Scot*t et a*l. 2012). Very high levels of disturbance may have undesirable consequences, though unstudied.

*Creation of permeable barriers*

1. The creation of permeable barriers, diversion ponds and artificial wetlands in small river catchments have well established effects on water flow (Richte*r et a*l. 2003; Wilkinson, Quinn & Welton 2010; Nicholso*n et a*l. 2012). However the benefits to biodiversity are unexplored. No obvious risks if applied carefully.

*Drainage control*

1. Blocking drainage ditches increases water availability in wetlands, leading to higher cranefly abundance in dry years (Carrol*l et a*l. 2011), though no significant changes in wet years. No adverse effects have been reported or are likely.
2. Creating artificial drains to divert water can provide better management of wet features. This allows control of invertebrate abundance location, improving lapwing chick body condition through increased food provision (Eglingto*n et a*l. 2008, 2010). Wet features are already present in the landscape and predation rates are unaffected through this technique (Eglingto*n et a*l. 2009)

*Irrigation/Spraying*

1. Irrigation/spraying increases in-situ water availability. This has been shown to increase mating and spawning in *Pseudophryne bibronii* Günther (brown toadlet) in remnant eucalyptus forest, South-east Australia (Mitchell 2001; Sho*o et a*l. 2011). Economically it is only feasible in a few areas, which limits its applicability. Krajick (2006) shows that the installation of a sprinkler system to mitigate against reduced river flow was insufficient to prevent the Kihansi spray toad *(Nectophrynoides asperginis*) from declined rapidly in Tanzania.

**Sea-level rise**

*Sea-defence maintenance / creation*

1. Hard defences can attenuate wave strength, as well as fragmenting coastal habitats leading to increases in local biodiversity (Airoldi et al. 2005). They may also improve connectivity between rocky coast regions. Financial costs may result in only economically important areas being maintained (Richards et al. 2008) and hard defences may reduce environmental heterogeneity, leading to less diverse intertidal communities (Firth et al. 2013).
2. Creation of artificial rockpools and increasing surface texture on sea defences leads to an increased diversity of algae and sessile and mobile invertebrates (Chapman & Blockley 2009; Firt*h et a*l. 2013, 2014). There is no obvious risk associated with this management as it is unlikely to negatively impact rocky shore organisms though benefits may be influenced by substrate type (Green, Chapman & Blockley 2012).
3. Options for creating ecologically-engineered offshore reefs, reducing wave energy. Oysters quickly colonise cultch material (shucked oyster shells) in high and low energy environments (Piazza, Banks & La Peyre 2005). Borsj*e et a*l. (2011) warn that it is important to understand habitat requirements for species settlement before applying this method to ensure it isn’t wasted effort.
4. Oyster and reef beds reduce wave energy (Borsj*e et a*l. 2011) and reduce shoreline erosion (Meyer, Townsend & Thayer 1997; Piazza, Banks & La Peyre 2005). This change is most beneficial in wave-exposed areas but these are most difficult conditions to establish reefs (Piazza, Banks & La Peyre 2005)
5. Groynes and other barriers provide stabilisation and/or accretion of material on sandy beaches and dunes. This is a widely-used management practice (Hanle*y et a*l. 2014) with high success rate, though may result in greater erosion elsewhere through loss of typical sediment.

*Stabilisation of coastal habitat*

1. Pumping of material from sediment sinks or dredging onto intertidal habitats increases the surface elevation in relation to sea level on salt marshes and sandy beaches. This leads to increased salt marsh plant biomass documented (Mendelssohn & Kuhn 2003) with the caveat that potential mineral deposition is an additional cause in this increase, as opposed to solely being the result of elevation increase. Also the source of sediment is important, since removal from the system can increase erosion elsewhere. Fine-grained material is more likely to be washed away (Hanle*y et a*l. 2014).
2. Planting of ecosystem-engineering species to stabilise sediment is another widely used method for stabilisation and/or accretion of habitat. Marram grass is widely planted to stabilise dune systems, even where the species in not native (Webb, Oliver & Pik 2000). Common cordgrass was widely planted in Europe for its ability to accrete sediment (Nehring & Hesse 2008). There can be high risk associated with this method where the species is not native, Common cordgrass is considered one of the top invasive species globally (Low*e et a*l. 2000).

*Defence realignment*

1. Saline inundation results in mudflat creation, providing suitable foraging habitat for wintering waterbirds (Atkinso*n et a*l. 2004; Badley & Allcorn 2006). Where tested, wintering waterbird community is similar to natural environments. However, where sedimentation rates are high, a shift from mudflat to salt marsh reduces suitability for feeding waders (Mazi*k et a*l. 2010; Morris 2013).
2. There is a rapid colonisation of new intertidal areas by a range of benthic invertebrate species (Mazi*k et a*l. 2010) though this is unlikely to provide a long-term fix. There are differences in abundance, diversity and community structure between a number of created and natural intertidal habitats (Craf*t et a*l. 1999; Mazi*k et a*l. 2010).
3. Newly created salt marsh allows for rapid establishment of some plant species (Mossma*n et a*l. 2012). Yet at some sites <50% of target species had established after 5 years (Wolter*s et a*l. 2008). Also characteristic perennials are rare in older, accidently realigned sites (Mossman, Davy & Grant 2012). This may in part be because it is difficult to replicate the geomorphological and topographic heterogeneity of natural salt marshes (Verbeek & Storm 2001; Doherty & Zedler 2015).

*Management of newly created intertidal habitat*

1. Planting rarer species on salt marshes increases the diversity of plant communities. Recruitment of individuals following planting can be high (Zedler, Callaway & Sullivan 2001) but is dependent on the species planted. Specifically survival is low if environmental conditions are not suitable for the species in question (Garbut*t et a*l. 2006).
2. Increasing topographic heterogeneity by creating raised and lowered areas on a salt marsh increases the range of available niches. Survival and recruitment of plants varies with raising and lowering treatments (Varty & Zedler 2008; Doherty & Zedler 2015). There is little risk associated with this method, though management must be carried out prior to instatement of tidal flooding due to accessibility for earth-moving equipment.

**References**

Airoldi, L., Abbiati, M., Beck, M.W., Hawkins, S.J., Jonsson, P.R., Martin, D., Moschella, P.S., Sundelöf, A., Thompson, R.C. & Åberg, P. (2005) An ecological perspective on the deployment and design of low-crested and other hard coastal defence structures. *Coastal Engineering*, **52**, 1073–1087.

Ashcroft, M.B., Chisholm, L.A. & French, K.O. (2009) Climate change at the landscape scale: predicting fine-grained spatial heterogeneity in warming and potential refugia for vegetation. *Global Change Biology*, **15**, 656–667.

Atkinson, P.W., Crooks, S., Drewitt, A., Grant, A., Rehfisch, M.M., Sharpe, J. & Tyas, C.J. (2004) Managed realignment in the UK - the first 5 years of colonization by birds. *Ibis*, **146**, 101–110.

Badley, J. & Allcorn, R. (2006) Changes in bird use following the managed realignment at Freiston Shore RSPB Reserve, Lincolnshire, England. *Conservation Evidence*, **3**, 102–105.

Bennie, J., Hill, M.O., Baxter, R. & Huntley, B. (2006) Influence of slope and aspect on long-term vegetation change in British chalk grasslands. *Journal of Ecology*, **94**, 355–368.

Bennie, J., Hodgson, J.A., Lawson, C.R., Holloway, C.T.R., Roy, D.B., Brereton, T., Thomas, C.D. & Wilson, R.J. (2013) Range expansion through fragmented landscapes under a variable climate. *Ecology Letters*, **16**, 921–929.

Bennie, J., Huntley, B., Wiltshire, A., Hill, M.O. & Baxter, R. (2008) Slope, aspect and climate: Spatially explicit and implicit models of topographic microclimate in chalk grassland. *Ecological Modelling*, **216**, 47–59.

Borsje, B.W., van Wesenbeeck, B.K., Dekker, F., Paalvast, P., Bouma, T.J., van Katwijk, M.M. & de Vries, M.B. (2011) How ecological engineering can serve in coastal protection. *Ecological Engineering*, **37**, 113–122.

Broadmeadow, S.B., Jones, J.G., Langford, T.E.L., Shaw, P.J. & Nisbet, T.R. (2011) The influence of riparian shade on lowland stream water temperatures in southern England and their viability for brown trout. *River Research and Applications*, **27**, 226–237.

Broadmeadow, S. & Nisbet, T.R. (2004) The effects of riparian forest management on the freshwater environment: a literature review of best management practice. *Hydrology and Earth System Sciences*, **8**, 286–305.

Carroll, M.J., Dennis, P., Pearce-Higgins, J.W. & Thomas, C.D. (2011) Maintaining northern peatland ecosystems in a changing climate: effects of soil moisture, drainage and drain blocking on craneflies. *Global Change Biology*, **17**, 2991–3001.

Chapman, M.G. & Blockley, D.J. (2009) Engineering novel habitats on urban infrastructure to increase intertidal biodiversity. *Oecologia*, **161**, 625–635.

Craft, C., Reader, J., Sacco, J.N. & Broome, S.W. (1999) Twenty-five years of ecosystem development of constructed *Spartina alterniflora* (Loisel) marshes. *Ecological Applications*, **9**, 1405–1419.

Davies, Z.G., Wilson, R.J., Coles, S. & Thomas, C.D. (2006) Changing habitat associations of a thermally constrained species, the Silver-spotted skipper butterfly, in response to climate warming. *Journal of Animal Ecology*, **75**, 247–256.

Doherty, J.M. & Zedler, J.B. (2015) Increasing substrate heterogeneity as a bet-hedging strategy for restoring wetland vegetation. *Restoration Ecology*, **23**, 15–25.

Eglington, S.M., Bolton, M., Smart, M.A., Sutherland, W.J., Watkinson, A.R. & Gill, J.A. (2010) Managing water levels on wet grasslands to improve foraging conditions for breeding northern lapwing *Vanellus vanellus*. *Journal of Applied Ecology*, **47**, 451–458.

Eglington, S.M., Gill, J.A., Bolton, M., Smart, M.A., Sutherland, W.J. & Watkinson, A.R. (2008) Restoration of wet features for breeding waders on lowland grassland. *Journal of Applied Ecology*, **45**, 305–314.

Eglington, S.M., Gill, J. A., Smart, M. A., Sutherland, W.J., Watkinson, A.R. & Bolton, M. (2009) Habitat management and patterns of predation of northern lapwings on wet grasslands: the influence of linear habitat structures at different spatial scales. *Biological Conservation*, **142**, 314–324.

Ellis, W., Melzer A., Clifton, I. & Carrick, F. (2010) Climate change and the koala Phascolarctos cinereus: water and energy. *Australian Zoologist*, **35**, 369-377.

Firth, L.B., Mieszkowska, N., Thompson, R.C. & Hawkins, S.J. (2013) Climate change and adaptational impacts in coastal systems: the case of sea defences. *Environmental Science: Processes & impacts*, **15**, 1665–1670.

Firth, L.B., Thompson, R.C., Bohn, K., Abbiati, M., Airoldi, L., Bouma, T.J., Bozzeda, F., Ceccherelli, V.U., Colangelo, M.A., Evans, A., Ferrario, F., Hanley, M.E., Hinz, H., Hoggart, S.P.G., Jackson, J.E., Moore, P., Morgan, E.H., Perkol-Finkel, S., Skov, M.W., Strain, E.M., van Belzen, J. & Hawkins, S.J. (2014) Between a rock and a hard place: environmental and engineering considerations when designing coastal defence structures. *Coastal Engineering*, **87**, 122–135.

Garbutt, R.A., Reading, C.J., Wolters, M., Gray, A.J. & Rothery, P. (2006) Monitoring the development of intertidal habitats on former agricultural land after the managed realignment of coastal defences at Tollesbury, Essex, UK. *Marine Pollution Bulletin*, **53**, 155–164.

Green, D.S., Chapman, M.G. & Blockley, D.J. (2012) Ecological consequences of the type of rock used in the construction of artificial boulder-fields. *Ecological Engineering*, **46**, 1–10.

Hanley, M.E., Hoggart, S.P.G., Simmonds, D.J., Bichot, A., Colangelo, M.A., Bozzeda, F., Heurtefeux, H., Ondiviela, B., Ostrowski, R., Recio, M., Trude, R., Zawadzka-Kahlau, E. & Thompson, R.C. (2014) Shifting sands? Coastal protection by sand banks, beaches and dunes. *Coastal Engineering*, **87**, 136–146.

Hardwick, S.R., Toumi, R., Pfeifer, M., Turner, E.C., Nilus, R. & Ewers, R.M. (2015) The relationship between leaf area index and microclimate in tropical forest and oil palm plantation: forest disturbance drives changes in microclimate. *Agricultural and Forest Meteorology*, **201**, 187–195.

Haskell, D.E., Flaspohler, D.J., Webster, C.R. & Meyer, M.W. (2012) Variation in soil temperature, moisture, and plant growth with the addition of downed woody material on lakeshore restoration sites. *Restoration Ecology*, **20**, 113–121.

Lawson, C.R., Bennie, J.J., Thomas, C.D., Hodgson, J.A. & Wilson, R.J. (2012) Local and landscape management of an expanding range margin under climate change. *Journal of Applied Ecology*, **49**, 552–561.

Lowe, S., Browne, M., Boudjelas, S. & De Poorter, M. (2000) *100 of the World’s Worst Invasive Alien Species: A Selection from the Global Invasive Species Database*. Invasive Species Specialist Group, Auckland.

Maclean, I.M.D., Bennie, J.J., Scott, A.J. & Wilson, R.J. (2012) A high-resolution model of soil and surface water conditions. *Ecological Modelling*, **238**, 109–119.

Malcolm, I.A., Soulsby, C., Hannah, D.M., Bacon, P.J., Youngson, A.F. & Tetzlaff, D. (2008) The influence of riparian woodland on stream temperatures: implications for the performance of juvenile salmonids. *Hydrological Processes*, **22**, 968–979.

Mazik, K., Musk, W., Dawes, O., Solyanko, K., Brown, S., Mander, L. & Elliott, M. (2010) Managed realignment as compensation for the loss of intertidal mudflat: a short term solution to a long term problem? *Estuarine, Coastal and Shelf Science*, **90**, 11–20.

McCormick, D.P. & Harrison, S.S.C. (2011) Direct and indiretc effects of riparian canopy on juvenile Atlantic salmon, *Salmo salar,* and brown trout, *Salmo trutta,* in south-west Ireland. *Fisheries Management and Ecology,* **18,** 444-455.

Mendelssohn, I.A. & Kuhn, N.L. (2003) Sediment subsidy: Effects on soil-plant responses in a rapidly submerging coastal salt marsh. *Ecological Engineering*, **21**, 115–128.

Meyer, D.L., Townsend, E.C. & Thayer, G.W. (1997) Stabilization and erosion control value of oyster cultch for intertidal marsh. *Restoration Ecology*, **5**, 93–99.

Mitchell, N.J. (2001) Males call more from wetter nests: Effects of substrate water potential on reproductive behaviours of terrestrial toadlets. *Proceedings of the Royal Society of London B: Biological Sciences*, **268**, 87–93.

Moore, R.D., Spittlehouse, D.L. & Story, A. (2005) Riparian microclimate and stream temperature response to forest harvesting: a review. *Journal of the American Water Resources Association*, **41**, 813–834.

Moreira, F. & Russo, D. (2007) Modelling the impact of agricultural abandonment and wildfires on vertebrate diversity in Mediterranean Europe. *Landscape Ecology*, **22**, 1461–1476.

Morris, R.K.A. (2013) Managed realignment as a tool for compensatory habitat creation - a re-appraisal. *Ocean and Coastal Management*, **73**, 82–91.

Mossman, H.L., Brown, M.J.H., Davy, A.J. & Grant, A. (2012) Constraints on salt marsh development following managed coastal realignment: Dispersal limitation or environmental tolerance? *Restoration Ecology*, **20**, 65–75.

Mossman, H.L., Davy, A.J. & Grant, A. (2012) Does managed coastal realignment create saltmarshes with ‘equivalent biological characteristics’ to natural reference sites? *Journal of Applied Ecology*, **49**, 1446–1456.

Nehring, S. & Hesse, K.J. (2008) Invasive alien plants in marine protected areas: The Spartina anglica affair in the European Wadden Sea. *Biological Invasions*, **10**, 937–950.

Nicholson, A.R., Wilkinson, M.E., O’Donnell, G.M. & Quinn, P.F. (2012) Runoff attenuation features: a sustainable flood mitigation strategy in the Belford catchment, UK. *Area*, **44**, 463–469.

Owens A.K., Moseley K.R., McCay T.S., Castleberry S.B., Kilgo J.C. & Ford W.M. (2008) Amphibian and reptile community response to coarse woody debris manipulations in upland loblolly pine (Pinus taeda) forests. *Forest Ecology and Management*, **256**, 2078-2083

Patrick D.A., Hunter M.L. & Calhoun A.J.K. (2006) Effects of experimental forestry treatments on a Maine amphibian community*. Forest Ecology and Management*, **234**, 323-332.

Parkyn, S.M., Davies‐Colley, R.J., Halliday, N.J., Costley, K.J., & Croker, G.F. (2003) Planted riparian buffer zones in New Zealand: do they live up to expectations? *Restoration Ecology*, **11**, 436-447.

Piazza, B.P., Banks, P.D. & La Peyre, M.K. (2005) The potential for created oyster shell reefs as a sustainable shoreline protection strategy in Louisiana. *Restoration Ecology*, **13**, 499–506.

Pyke, C.R. & Marty, J. (2005) Cattle grazing mediates climate change impacts on ephemeral wetlands. *Conservation Biology*, **19**, 1619–1625.

Richards, J.A., Mokrech, M., Berry, P.M. & Nicholls, R.J. (2008) Regional assessment of climate change impacts on coastal and fluvial ecosystems and the scope for adaptation. *Climatic Change*, **90**, 141–167.

Richter, B.D., Mathews, R., Harrison, D.L. & Wigington, R. (2003) Ecologically sustainable water management: managing river flows for ecological integrity. *Ecological Applications*, **13**, 206–224.

Scheffers, B.R., Edwards, D.P., Diesmos, A., Williams, S.E. & Evans, T.A. (2014a) Microhabitats reduce animal’s exposure to climate extremes. *Global Change Biology*, **20**, 495–503.

Scheffers, B.R., Evans, T.A., Williams, S.E. & Edwards, D.P. (2014b) Microhabitats in the tropics buffer temperature in a globally coherent manner. *Biology Letters*, **10**.

Scott, A., Maclean, I.M.D., Byfield, A., Pay, A.R. & Wilson, R.J. (2012) Artificial disturbance promotes recovery of rare Mediterranean temporary pond plant species on the Lizard peninsula, Cornwall, England. *Conservation Evidence*, **9**, 79–86.

Semlitsch R.D., Todd B.D., Blomquist S.M., Calhoun A.J.K., Whitfield-Gibbons J., Gibbs J.P., Graeter G.J., Harper E.B., Hocking D.J., Hunter M.L., Patrick D.A., Rittenhouse T.A.G. & Rothermel B.B. (2009) Effects of timber harvest on amphibian populations: understanding mechanisms from forest experiments. *BioScience*, **59**, 853-862

Shoo, L.P., Olson, D.H., McMenamin, S.K., Murray, K.A., Van Sluys, M., Donnelly, M.A., Stratford, D., Terhivuo, J., Merino-Viteri, A., Herbert, S.M., Bishop, P.J., Corn, P.S., Dovey, L., Griffiths, R.A., Lowe, K., Mahony, M., McCallum, H., Shuker, J.D., Simpkins, C., Skerratt, L.F., Williams, S.E. & Hero, J.-M. (2011) Engineering a future for amphibians under climate change. *Journal of Applied Ecology*, **48**, 487–492.

Suggitt, A.J., Stefanescu, C., Paramo, F., Oliver, T., Anderson, B.J., Hill, J.K., Roy, D.B., Brereton, T. & Thomas, C.D. (2012) Habitat associations of species show consistent but weak responses to climate. *Biology Letters*, **8**, 590–593.

Suggitt, A.J., Wilson, R.J., August, T.A., Beale, C.A., Bennie, J.J., Dordolo, A., Fox, R., Hopkins, J.J., Isaac, N.J.B., Jorieux, P., MacGregor, N.A., Marcetteau, J., Massimino, D., Morecroft, M.D., Pearce-Higgins, J.W., Walker, K. & Maclean, I.M.D. (2014) *Climate Change Refugia for the Flora and Fauna of England*. Natural England Commissoned Reports, Number 162. Natural England, York.

Suggitt, A.J., Wilson, R.J., August, T.A., Fox, R., Isaac, N.J.B., Macgregor, N. a, Morecroft, M.D. & Maclean, I.M.D. (2015) Microclimate affects landscape level persistence in the British Lepidoptera. *Journal of Insect Conservation*, **19**, 237–253.

Thomas, J.A., Simcox, D.J., Wardlaw, J.C., Elmes, G.W., Hochberg, M.E. & Clarke, R.T. (1998) Effects of latitude, altitude and climate on the habitat and conservation of the endangered butterfly *Maculinea arion* and its Myrmica ant hosts. *Journal of Insect Conservation*, **2**, 39–46.

Varty, A.K. & Zedler, J.B. (2008) How waterlogged microsites help an annual plant persist among salt marsh perennials. *Estuaries and Coasts*, **31**, 300–312.

Verbeek, H. & Storm, C. (2001) Tidal wetland restoration in the Netherlands. *Journal of Coastal Research*, **SI**, 192–202.

Wanger T.C., Saro A., Iskandar D.T., Brook B.W., Sodhi N.S., Clough Y. & Tscharntke T. (2009) Conservation value of cacao agroforestry for amphibians and reptiles in South-East Asia: combining correlative models with follow-up field experiments. *Journal of Applied Ecology*, **46**, 823-832.

Webb, C.E., Oliver, I. & Pik, A.J. (2000) Does coastal foredune stabilization with *Ammophila arenaria* restore plant and arthropod communities in southeastern Australia? *Restoration Ecology*, **8**, 283–288.

Whitledge, G.W., Rabeni, C.F., Annis, G. & Sowa, S.P. (2006) Riparian shading and groundwater enhance growth potential for smallmouth bass in ozark streams. *Ecological Applications*, **16**, 1461–1473.

Wilkinson, M.E., Quinn, P.F. & Welton, P. (2010) Runoff management during the September 2008 floods in the Belford catchment, Northumberland. *Journal of Flood Risk Management*, **3**, 285–295.

Wolters, M., Garbutt, A., Bekker, R.M., Bakker, J.P. & Carey, P.D. (2008) Restoration of salt-marsh vegetation in relation to site suitability, species pool and dispersal traits. *Journal of Applied Ecology*, **45**, 904–912.

Zedler, J.B., Callaway, J.C. & Sullivan, G. (2001) Declining biodiversity: why species matter and how their functions might be restored in Californian tidal marshes. *BioScience*, **51**, 1005.
