# Supplementary material for: Using in situ management to conserve biodiversity under climate change
Source: J Appl Ecol. 2016 Jan 19;53(3):885–94. doi: 10.1111/1365-2664.12602 (PMC4991270; doi:10.1111/1365-2664.12602)
Supplement: Supplementary file 6 — Appendix S3. Additional information and references associated with Table 2. [file JPE-53-885-s006.docx]

**Appendix S3: Additional information and references associated with Table 3**

^1^Longer vegetation could potentially provide species with cooler microhabitats. Five studies (Willems, Peet & Bik 1993; Van der Woude, Pegtel & Bakker 1994; Foster & Gross 1998; Hejcman *et al.* 2007; Honsova *et al.* 2007) show that the experimental application of nitrogen, phosphorus and/or potassium, while increasing productivity, reduced the species richness of grasslands. Numerous studies demonstrate adverse effects on species richness of nutrient enrichment and demonstrate benefits to biodiversity of reduced fertilizer application.

^2^Retaining areas of flooded habitat may benefit species in locations predicted to become drier. A study of rice fields in southern Brazil (Machado & Maltchik 2010) found that keeping fields flooded after harvest had no effect on amphibian species richness or abundance

^3^Wet habitats have been lost from agricultural systems, a problem compounded in regions predicted to become drier. Re-wetting may offer an important mechanism for offsetting this effect, facilitating a reverse in declines. A review of three studies by Diggelen (2007) shows that re-wetting soils on old arable fields is not an effective method of restoring species-rich grassland.

^4^In habitats prone to sea-level rise, inundation with water may be a significant cause of mortality. Providing nests or nesting habitat that is protected from water (e.g. by being raised) may therefore increase reproductive success. Two studies from the USA (Koenen *et al.* 1996; Rounds *et al.* 2004) found that the nesting success of terns and waders was no higher on specially raised areas of nesting substrate, compared to unraised areas.

**References**

Diggelen, R.V. (2007) Habitat creation: nature conservation of the future? *Aspects of Applied Biology*, **82**, 1-11

Foster, B.L. & Gross, K.L. (1998) Species richness in a successional grassland: effects of nitrogen enrichment and plant litter. *Ecology*, **79**, 2593-2602.

Hejcman, M., Klaudisová, M., Schellberg, J., & Honsová, D. (2007) The Rengen Grassland Experiment: plant species composition after 64 years of fertilizer application. *Agriculture, Ecosystems & Environmen*t, **122**, 259-266.

Machado, I.F. & Maltchik, L. (2010) Can management practices in rice fields contribute to amphibian conservation in southern Brazilian wetlands? *Aquatic Conservation*, **20**, 39-46

Koenen, M.T., Utych, R.B. & Leslie, D.M. (1996) Methods used to improve least tern and snowy plover nesting success on alkaline flats. *Journal of Field Ornithology*, **67**, 281-291

Krajick, K. (2006) The lost world of the Kihansi toad. *Science*, **311**, 1230–1232

Honsova, D., Hejcman, M., Klaudisova, M., Pavlu, V., Kocourkova, D., & Hakl, J. (2007). Species composition of an alluvial meadow after 40 years of applying nitrogen, phospohorus and potassium fertilizer. *Preslia-Praha* **79**, 245-258.

Rounds, R.A., Erwin, R.M. & Porter, J.H. (2004) Nest-site selection and hatching success of waterbirds in coastal Virginia: some results of habitat manipulation. *Journal of Field Ornithology,* **75**, 317-329.

Van der Woude, B.J., Pegtel, D.M., & Bakker, J.P. (1994). Nutrient limitation after long-term nitrogen fertilizer application in cut grasslands. *Journal of Applied Ecology*, **31**, 405-412.

Willems, J.H., Peet, R.K. & Bik, L. (1993) Changes in chalk‐grassland structure and species richness resulting from selective nutrient additions. *Journal of Vegetation Science*, **4**, 203-212.
